# Supplementary material for: ERGA-BGE genome of Stigmatoteuthis arcturi Robson, 1948: the jewelled squid
Source: Open Res Eur. 2025 Sep 4;5:270. [Version 1] doi: 10.12688/openreseurope.20439.1 (PMC12475895; doi:10.12688/openreseurope.20439.1)
Supplement: Peer review history

# Reviewer report: Reviewer response for version 1

**DOI:** 10.21956/openreseurope.22118.r60082

**Published:** 2025-10-08

**Authors:** Marco Gerdol

**Affiliations:** 1 University of Trieste, Trieste, Italy

The manuscript by Fernandez-Alvarez and colleagues reports the genome assembly of *Stigmatoteuthis arcturi*, a deep sea squid. Although the approach is technically sound and gold-standard methodologies were used to generate raw sequencing data and to obtain the genome assembly and annotation, a number of points could be slightly improved for the sake of clarity.

 Materials and methods: the introductory section may be removed. In understand that this might be a standard sentence recommended for the all ERGA genomes, but in this case non PacBio sequencing was used, for example, and therefore this information, placed here, is somewhat misleading.

 "As the squid is not considered an experimental animal" is unclear. Please explain what does this mean. I guess that a blue whale would not be considered an "experimental animal" either, but I strongly doubt that no approval by an ethical committee would be needed in that case. Generally speaking, such approval are linked with the texonomical classification of a species, and regulations about cephalopods may vary from nation to nation. Please be more specific about this point, since the meaning of "experimental animal" is unclear.

 Genetic information: as far as I can see from the linked ERGA document, the expected haploid number of chromosomes was 6 (unless there is a mistake in the file), so 46 was the observed number of chromosomes after the assembly. If this was the case, then I don't see any reason for this section to exist or to be placed here: simply report these data later, when the genome assembly statistics are mentioned.

 Genome annotation methods: "a select set of clade-specific proteins from UniProt". Please aplain which criteria were used to select these proteins, with particular reference to the fact they were "clade-specific". This does not mean much, unless you explain which taxonomic rank was used to define the clade (e.g. Cephalopoda, Mollusca, etc.).

 Genome assembly: I would have expected here a clear statement about the fraction of the genome assembly size falling within the 46 expected chromosomes.

 The organization of Table 2 may look unclear to most readers. The caption should clarify that the consistency scores only apply to OMArk, and that they are linked with all predicted protein-coding gene models (unlink completeness evaluation, which only concerns a subset of genes).

 Data availability: a link the gene annotation track should be more explicitly provided in this section, since this is not available from the ERGA portal.

Are sufficient details of methods and materials provided to allow replication by others?

Yes

Is the rationale for creating the dataset(s) clearly described?

Yes

Are the datasets clearly presented in a useable and accessible format?

Partly

Are the protocols appropriate and is the work technically sound?

Partly

Reviewer Expertise:

Molluscan genomics

I confirm that I have read this submission and believe that I have an appropriate level of expertise to confirm that it is of an acceptable scientific standard, however I have significant reservations, as outlined above.

# Reviewer report: Reviewer response for version 1

**DOI:** 10.21956/openreseurope.22118.r60078

**Published:** 2025-09-27

**Authors:** José M Martín-Durán

**Affiliations:** 1 Queen Mary University of London, London, England, UK

This genome note reports the genome assembly and annotation of the jewelled squid *Stigmatoteuthis arcturi*. This is an important dataset and resource for the study of oceanic cephalopods that will benefit multiple research fields, from phylogenomics and evolutionary biology to ecology. Strengths: This is a gold-standard, high-quality resource. The genome assembly comes with an annotation, which will allow immediate re-use of the data; Weaknesses: none.

Are sufficient details of methods and materials provided to allow replication by others?

Yes

Is the rationale for creating the dataset(s) clearly described?

Yes

Are the datasets clearly presented in a useable and accessible format?

Yes

Are the protocols appropriate and is the work technically sound?

Yes

Reviewer Expertise:

Evo-Devo, comparative genomics and epigenomics

I confirm that I have read this submission and believe that I have an appropriate level of expertise to confirm that it is of an acceptable scientific standard.

# Reviewer report: Reviewer response for version 1

**DOI:** 10.21956/openreseurope.22118.r60081

**Published:** 2025-09-16

**Authors:** Masa-Aki Yoshida

**Affiliations:** 1 Shimane University, Shiman, Japan

Overall, the study is well documented and appears publishable; however, for the benefit of future related research, the following minor point should be addressed.

 As the authors themselves note, genomic analysis of pelagic squid remains limited and incomplete. Simply estimating genome size by comparison with other species is not acceptable. Although the assembled size appears consistent, both genome size and chromosome number should be independently validated using alternative methods.

Are sufficient details of methods and materials provided to allow replication by others?

Yes

Is the rationale for creating the dataset(s) clearly described?

Yes

Are the datasets clearly presented in a useable and accessible format?

Yes

Are the protocols appropriate and is the work technically sound?

Yes

Reviewer Expertise:

Bioinformatics, Genomics

I confirm that I have read this submission and believe that I have an appropriate level of expertise to confirm that it is of an acceptable scientific standard.
